# Transformation-associated recombination (TAR) cloning and its applications for gene function; genome architecture and evolution; biotechnology and biomedicine

**DOI:** 10.18632/oncotarget.28546

**Published:** 2023-12-22

**Authors:** Natalay Kouprina, Vladimir Larionov

**Affiliations:** ^1^Developmental Therapeutics Branch, National Cancer Institute, Bethesda, MD 20892, USA

**Keywords:** transformation-associated recombination, TAR, microbes, biomedicine, biotechnology

## Abstract

Transformation-associated recombination (TAR) cloning represents a unique tool to selectively and efficiently recover a given chromosomal segment up to several hundred kb in length from complex genomes (such as animals and plants) and simple genomes (such as bacteria and viruses). The technique exploits a high level of homologous recombination in the yeast *Sacharomyces cerevisiae*. In this review, we summarize multiple applications of the pioneering TAR cloning technique, developed previously for complex genomes, for functional, evolutionary, and structural studies, and extended the modified TAR versions to isolate biosynthetic gene clusters (BGCs) from microbes, which are the major source of pharmacological agents and industrial compounds, and to engineer synthetic viruses with novel properties to design a new generation of vaccines. TAR cloning was adapted as a reliable method for the assembly of synthetic microbe genomes for fundamental research. In this review, we also discuss how the TAR cloning in combination with HAC (human artificial chromosome)- and CRISPR-based technologies may contribute to the future.

## INTRODUCTION

TAR cloning is the method that enables selective, rapid, and efficient capture of genes or chromosomal regions of choice from total genomic DNA of organisms ranging from microbes and viruses to plants, and animals as circular YAC/BAC (yeast artificial chromosome/bacterial artificial chromosome) molecules which can propagate in yeast as well as in bacterial cells. TAR cloning exploits the efficient non-meiotic homologous recombination of the yeast *Saccharomyces cerevisiae* [1–4]. A desired region of choice is captured from genomic DNA using TAR vector containing either two unique sequences (hooks) homologous to the 5′- and 3′-ends of the target region (see chapter 3.1) or one unique hook and a common repeat (see chapter 3.3) or using a counter selectable marker (see chapter 3.2) [5–9].

**Figure 1:**
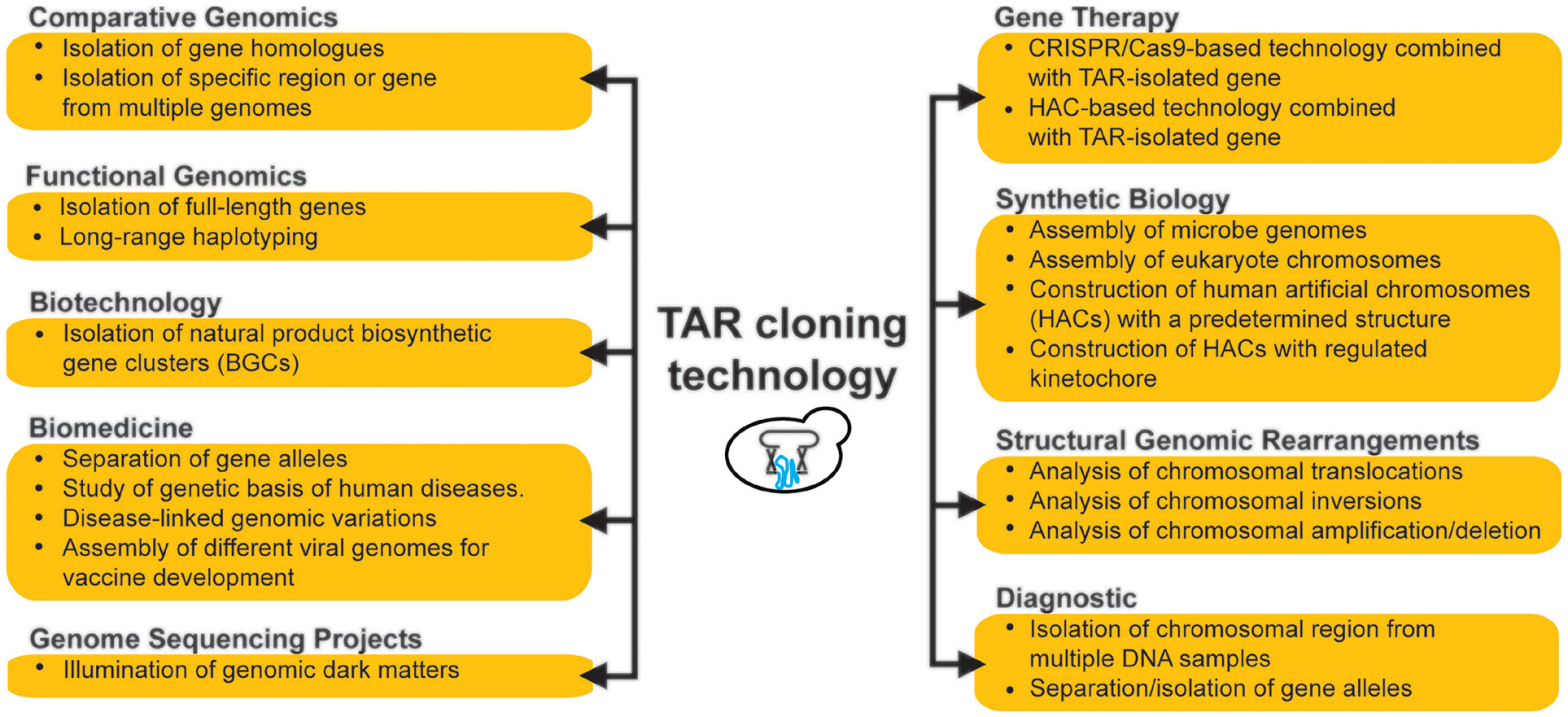
Applications of the TAR cloning technology.

For gene functional studies and potentially for gene therapy, it is important that TAR cloning isolates full-length genes that contain coding (exons) as well as non-coding (introns) regions, including their regulatory elements, that can reproduce a physiological gene expression. For the past two decades, TAR cloning has become a reliable method with multiple applications in functional and structural genomics, characterization of chromosomal rearrangements, evolutionary studies, and engineering synthetic microbial genomes and viruses with contribution in biotechnology and biomedicine (Figure 1).

Recently TAR cloning has been adapted and widely used to clone BGCs from microbes and environmental DNA samples (see chapter 4.4) [10–12]. Typically, natural products (NPs) BGCs are produced by co-expression of multiple genes involved in regulation, biosynthesis, transport, and resistance to the drug(s) [13]. A direct capture of intact BGCs by TAR cloning has proven to be important for discovery of novel NPs to understand their biosynthesis and molecular mechanisms [11].

The previous methods of isolation of mammalian genes and BGCs relied on the construction of YAC, BAC, or cosmid libraries followed by PCR screening of thousands of transformants to identify the ones of your interest. In such libraries, the frequency of region-positive clones was less than 0.0003% [14]. If we compare, for example, the efficiency of TAR cloning of a human gene (~35%), with the frequency of gene recovery from YAC or BAC libraries (0.0003%), TAR cloning efficiency is higher than ~100.000 times. Moreover, from the libraries a desired region is often recovered as a set of DNA fragments. In such cases, they need to be pieced together to reassemble the entire region that makes this method technically challenging and time-consuming.

An alternative technique, Cas9-Assisted Targeting of Chromosome segments (CATCH), has been developed and applied to recover BGCs from microorganisms directly in *E. coli* [15]. This method is based on the *in-vitro* cleavage of target DNA from a native bacterial chromosome using RNA-guided Cas9 nuclease and subsequent ligation into a cloning vector via Gibson assembly [16]. In principle, this method may be considered as an alternative to TAR cloning when the goal is to isolate BGC from the individual microbe. However, it has never been applied to isolate BGCs from environmental samples (e.g., from soil or gut microbiota) that typically contain thousands of bacterial species or from complex genomes such as animals and plants.

In this review, we focus on the main TAR cloning parameters, TAR cloning variations, and multiple applications, i.e., isolation of intact genes and gene clusters for functional, evolutionary, and structural studies and capture of intact BGCs for heterologous expression and natural product discovery. In addition, we describe the application of TAR for cloning and assembly of Mb-scale microbe genomes. Finally, we discuss the progress in applying the TAR technology to construct HACs for functional and structural studies of human kinetochore. The benefit of combining the TAR cloning technology with the HAC gene delivery system for therapeutic gene delivery and expression studies is also discussed.

## PARAMETERS OF TAR CLONING

### Features of TAR vector

A TAR vector contains a YAC cassette (a yeast selectable marker and a yeast centromere) for proper propagation, segregation, and selection of the cloned material in yeast, and a BAC cassette (a bacterial origin of replication and a bacterial selectable marker) that allows TAR isolates to propagate in bacterial cells. TAR vectors used for isolation of DNA fragments from complex genomes does not contain a yeast origin of replication and therefore cannot propagate in yeast cells producing no background. In this case, the TAR cloning method requires a presence of at least one autonomously replicating sequence (*ARS*) that can function as yeast origin of replication in the cloned genomic DNA fragment. Potential *ARS*-like sequences have a 17-bp *ARS* core consensus, WWWWTT TAYRTTTWGTT, in which W = A or T, Y = T or C, and R = A or G [17]. Such sequences occur at a frequency of approximately one per 20–40 kb in all eukaryotic genomes thus far examined [18]. This suggests that TAR cloning can readily isolate most chromosomal regions with a vector that lacks an *ARS* because it relies on the acquisition of an *ARS* element from the targeted chromosomal DNA fragment. Some genomic regions in microbes also contain *ARS*-like sequences and may be captured by TAR vector lacking *ARS*. So far, several hundred different genomic regions were isolated using TAR vectors without *ARS* [19]. Features of TAR vector used for isolation of DNA fragments from simple genomes, such as bacterial or viral, are described in chapter 3.2.

### Size and divergence requirements for the targeting sequences (hooks) in TAR vectors

**Figure 2:**
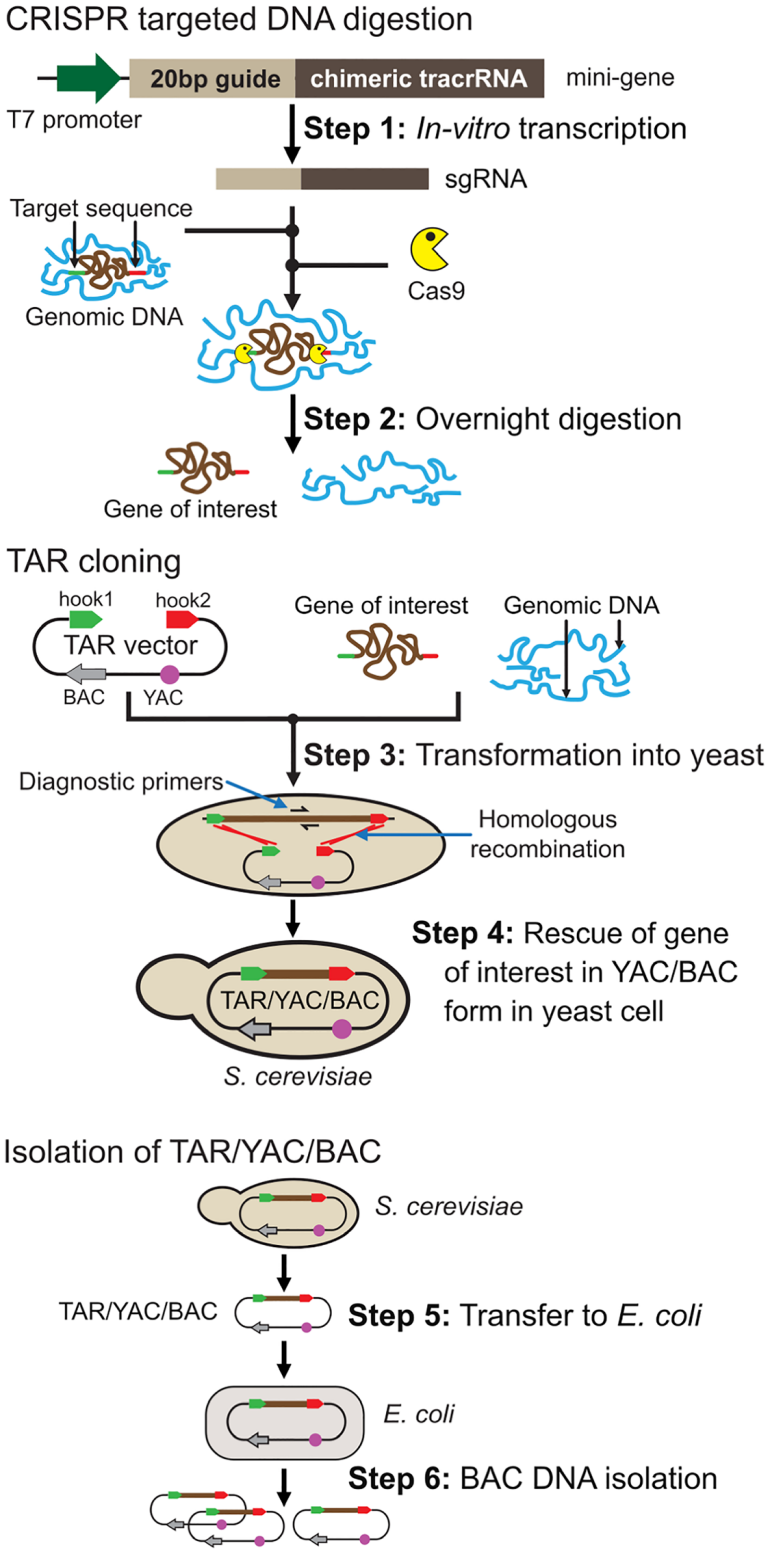
The experimental consecutive steps of TAR isolation of a gene/region of choice from the total genome. Preparation of genomic DNA for TAR cloning. **Step 1:** Genomic DNA is cleaved at the positions close to the 5′ and 3′ ends of a region of choice by Cas9-gRNA complexes. The CRISPR minigene may be generated by PCR or artificially synthesized. **Step 2:** Overnight digestion of the mixture containing gRNAs, Cas9 and genomic DNA. TAR cloning of a region of choice from genomic DNA. A TAR vector contains YAC (in purple) and BAC (in grey) cassettes and two unique targeting sequences (hook1 in green and hook2 in red) homologous to the 5′ (in green) and 3′ (in red) ends of a region of choice. Before cloning, TAR vector is linearized between the hooks to expose targeting hooks. **Step 3:** CRISPR/Cas9-treated genomic DNA and a linearized TAR vector are co-transformed into yeast *Saccharomyces cerevisiae* cells. **Step 4:** Recombination between targeting hooks in the vector and the target sequences in the genomic DNA region leads to rescue of the genomic region as a circular YAC/BAC molecule. (**c**) Isolation of YAC molecules containing a region of choice from yeast cells [148]. **Step 5:** Transferring TAR-isolated YAC/BAC molecules from yeast cells to bacterial cells. **Step 6:** BAC DNA is isolated for further sequencing and functional analyses.

A genomic region of choice is targeted by TAR vector containing two unique guiding sequences (hooks) homologous to the 5′ and 3′ flanks of the target. The minimal size of these hooks may be as short as 60 bp, though longer hooks can also be used [20]. Hooks should be unique sequences, which can be assured by blasting candidate sequences against a genome reference sequence (http://genome.ucsc.edu/cgi-bin/hgBlat). The hooks are cloned into TAR vector in the same orientation as they occur in the targeted genome. Prior to TAR cloning experiments, a vector is linearized between the hooks to make them highly recombinogenic (Figure 2).

It was found that a divergence of up to 15% between the targeting hooks and the target genomic sequences does not prevent isolation of specific genes or regions from different organisms using TAR vector with the hooks developed from the human genome sequence [21]. The yield of region-positive clones with TAR vector containing the unique sequence hooks is comparable to that when the hooks are divergent by 15%. Such robustness with respect to DNA sequence enables TAR cloning to be applied to isolate gene orthologs and paralogs.

### The size of TAR-isolated and TAR-assembled genomes and genomic regions

So far, TAR cloning allowed isolating genomic fragments up to 300 kb in size, which is sufficient for successful cloning of most mammalian genes and microbial gene clusters. However, this limit is not absolute, as some YAC libraries contain YACs ranging from 430 kb to 1,200 kb [22]. To TAR-isolate a genomic DNA fragment with the size bigger than 50 kb, DNA should be protected from shearing by preparing a high-molecular weight DNA in agarose blocks [9]. Note that after isolation of a region of choice in yeast, if necessary, this region may be easily modified using homologous recombination in yeast [23, 24] or transferred directly to another host, for example bacterial cells.

As described, there is almost no size limitation for the genomic fragment to propagate in yeast cells. Assembly of individual chromosomes or whole genomes by transformation-associated recombination in yeast was successfully accomplished for species such as *M. pneumoniae* (0.8 Mb) [25], *Acholeplasma laidlawii* (1.5 Mb) [26], *Prochlorococcus marinus MED4* (1.6 Mb) [27], and eukaryotic algal *Phaeodactylum tricornutum* (27.4 Mb) [28]. In the latter case, two individual chromosomes were assembled using TAR. Recently, several groups assembled the entire viral genomes using a TAR platform [29, 30].

### Anticipated results

Once TAR vector with its specific hooks is constructed and genomic DNA is prepared, the entire procedure to isolate a gene or a region of choice takes approximately three weeks. With 0.5–1 mg of TAR vector, 1–2 mg of genomic DNA and 1 × 10^8^ yeast spheroplasts, the yield of transformants varies from 10 to 150 colonies on one Petri dish. The yield of region-positive clones from mammalian or individual microbial genomes or environmental DNA samples varies from 35% to 95% [20]. In the case of complex genomes, it is preferrable that genomic DNA is pre-treated before spheroplast transformation by CRISPR-Cas9 (clustered regularly interspaced short palindromic repeats) that are recognized by Cas9 nuclease [31–35] that is designed in such a way to cut near the targeted 5′ and 3′ end sequences making them highly recombinogenic [9, 36]. In this case, for example, the yield of region-positive clones isolated from complex genomes increases from 1% up to 35 % [36].

## VARIATIONS OF TAR CLONING APPROACHES

### CRISPR/Cas9-mediated TAR cloning using a vector with two unique targeting hooks

For the past decade, the cost, quality, and efficacy of the TAR cloning method has been improved significantly by rigorous testing for the accuracy of isolation of loci from different organisms, shifting the technology from unusual to routine. The updated TAR protocol does not require significant experience with yeast, because screening of approximately 20–30 yeast transformants is typically enough to find a clone containing the region of choice [9]. Figure 2 shows a step-by step general scheme of TAR cloning of a region of choice from genomic DNA using TAR vector containing two targeting hooks [9]. Genomic DNA is pre-treated with the specifically designed programmable endonuclease CRISPR/Cas9 (Step 1 and Step 2 in Figure 2) that creates double-strand breaks (DSBs) bracketing the target genomic DNA sequence leading to increase of region-positive clones 35 times [9, 36]. Step 3 and Step 4 in Figure 2 include co-transformation of TAR vector linearized between the hooks and genomic DNA into competent yeast spheroplasts followed by recombination between the target hook sequences in the vector and targeted 3′ and 5′ ends of a genomic segment and rescue of a region of choice as a circular YAC/BAC molecule. Selection of region-positive clones is carried out by PCR using diagnostic primers. The TAR-cloned material may be directly moved from yeast cells to bacterial cells (Step 5) that facilitates BAC DNA isolation (Step 6 in Figure 2) for a further analysis.

### TAR cloning using a counter-selectable marker

**Figure 3:**
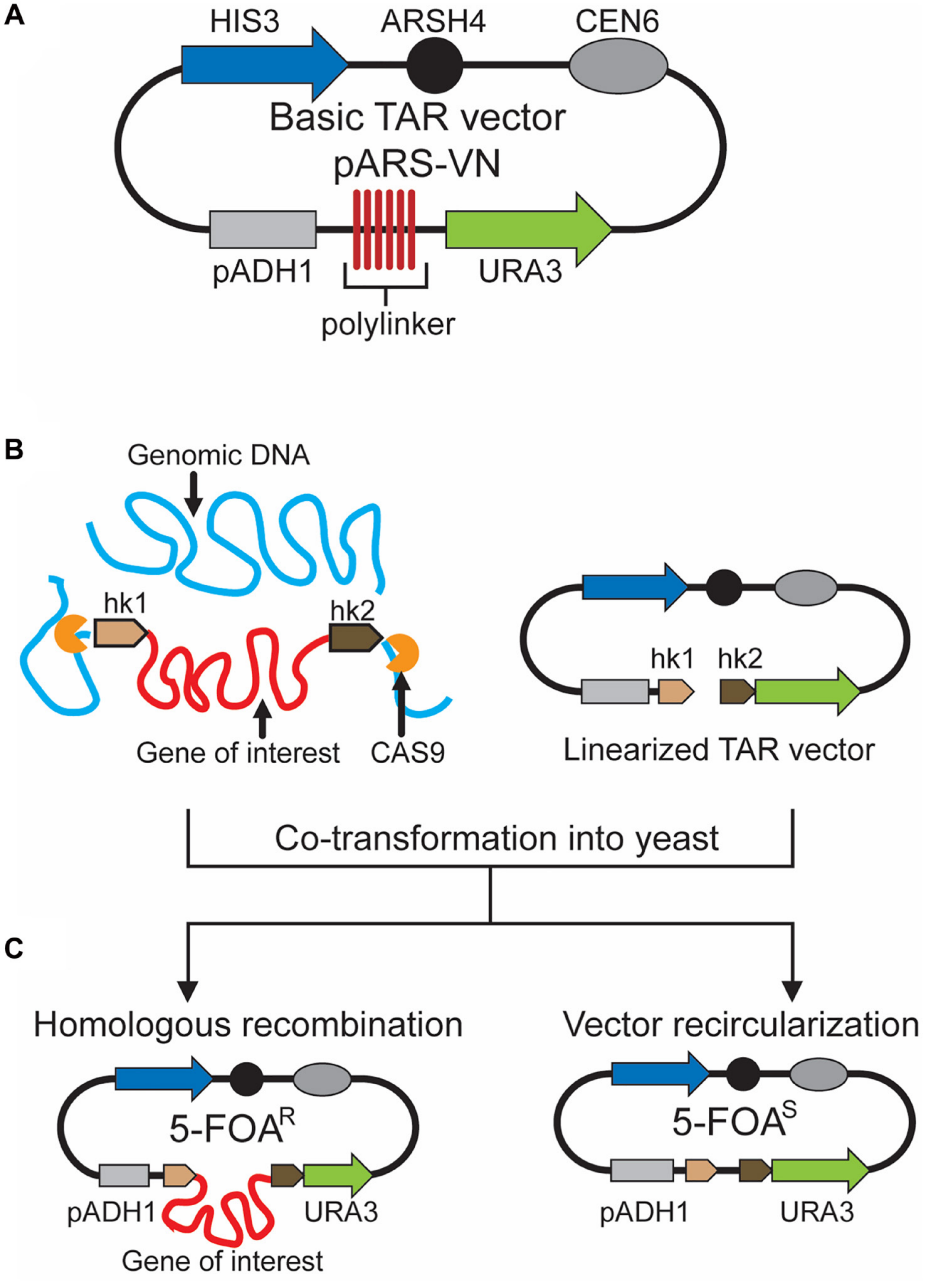
TAR cloning of genomic regions lacking *ARS* sequences from microbial and viral genomes. (**A**) Scheme of the basic TAR vector pARS-VN. The plasmid carries a yeast positive-selectable marker (*HIS3*) (in blue), a yeast centromere (*CEN6*) (in dark grey), a negative-selectable marker (*URA3*) (in green), a pADH1 promoter of the *ADH1* gene (in light grey), a yeast origin of replication (*ARSH4*) (in black), and a polylinker (a multiple cloning site) (in red). *URA3* is a hybrid gene containing the open reading frame of the *S. cerevisiae URA3* gene and the promoter of the *S. pombe pADH1* gene, which only tolerates insertion of up to 130 bp of sequence between the TATA box and the transcription initiation site. The combined length of the targeting hooks should be less than 130 bp in order not to disrupt *URA3* expression. Such configuration allows selection of TAR cloning events rather than vector recircularization. (**B**, **C**) A diagram of TAR cloning of a region of choice (in red) from genomic DNA (in blue). A TAR vector contains two unique targeting hooks (hk1 in beige and hk2 in brown) homologous to the 5′ and 3′ ends of a region of choice. The TAR vector DNA is linearized between the hooks to expose targeting sequences. If necessary, genomic DNA may be treated by CRISPR-Cas9 endonuclease before yeast transformation [36] to increase the yield of region-positive clones. Homologous recombination between the hooks and the target genomic sequence leads to insertion of the cloned material between the TATA box and the transcription initiation site ATG, which abolishes *URA3* expression. Such clones can, therefore, be selected by their ability to grow on media containing 5-FOA (5-FOA^R^). Recircularization of TAR vector alone due to nonhomologous end-joining leads to reconstruction of the counter-selectable *URA3* marker. In this case, the distance between the TATA element and the transcription initiation site ATG is not changed, and the *URA3* marker is normally expressed. Therefore, they do not grow on media containing 5-FOA (5-FOA^S^). (R = resistant, S = sensitive to 5-FOA).

For chromosomal GC-rich regions such as centromeres and telomeres of mammalian genomes or simple genomes like microbes and viruses that have few or no *ARS*-like sequences, the TAR method described in chapter 3.1 is not applicable. To overcome that limitation, a substantially different version of the TAR method has been developed [10–12]. A general scheme of this version using a counter-selectable marker is shown in Figure 3. The TAR vector contains a yeast *ARS* element and a negative-selectable marker *URA3* that represents a hybrid gene containing the open reading frame of the *S. cerevisiae URA3* gene and the promoter of the *S. pombe ADH1* gene (Figure 3A), which has strict spacing requirements for its function, i.e., the distance between the TATA element of the promoter and the transcription initiation site must be no more than 130 bp [37, 38]. Accordingly, the combined length of the targeting hooks in the TAR vector should not exceed 130 bp. The hooks are placed between the TATA box and the transcription initiation site of *URA3*. As a result of such a design, an insertion of any genomic fragment between the hooks due to homologous recombination between the hooks and the target genomic sequences (Figure 3B) leads to the inactivation of *URA3* expression (5-FOA^R^) (Figure 3C). Thus, because yeast cells expressing *URA3* are sensitive to 5-fluoroorotic acid (5-FOA^S^), the proper TAR clones containing a region of choice should be selected against the background (5-FOA^R^) arising from vector recircularization (5-FOA^S^) (Figure 3C). It is worth noting that sometimes TAR cloning of GC-rich bacterial DNA regions can be challenging even with the TAR *ARS*-containing vector. In these cases, only the fragments of approximately 100 kb or bigger are typically recovered [39].

### Radial TAR cloning using a vector with a unique targeting hook and a common repeat

**Figure 4:**
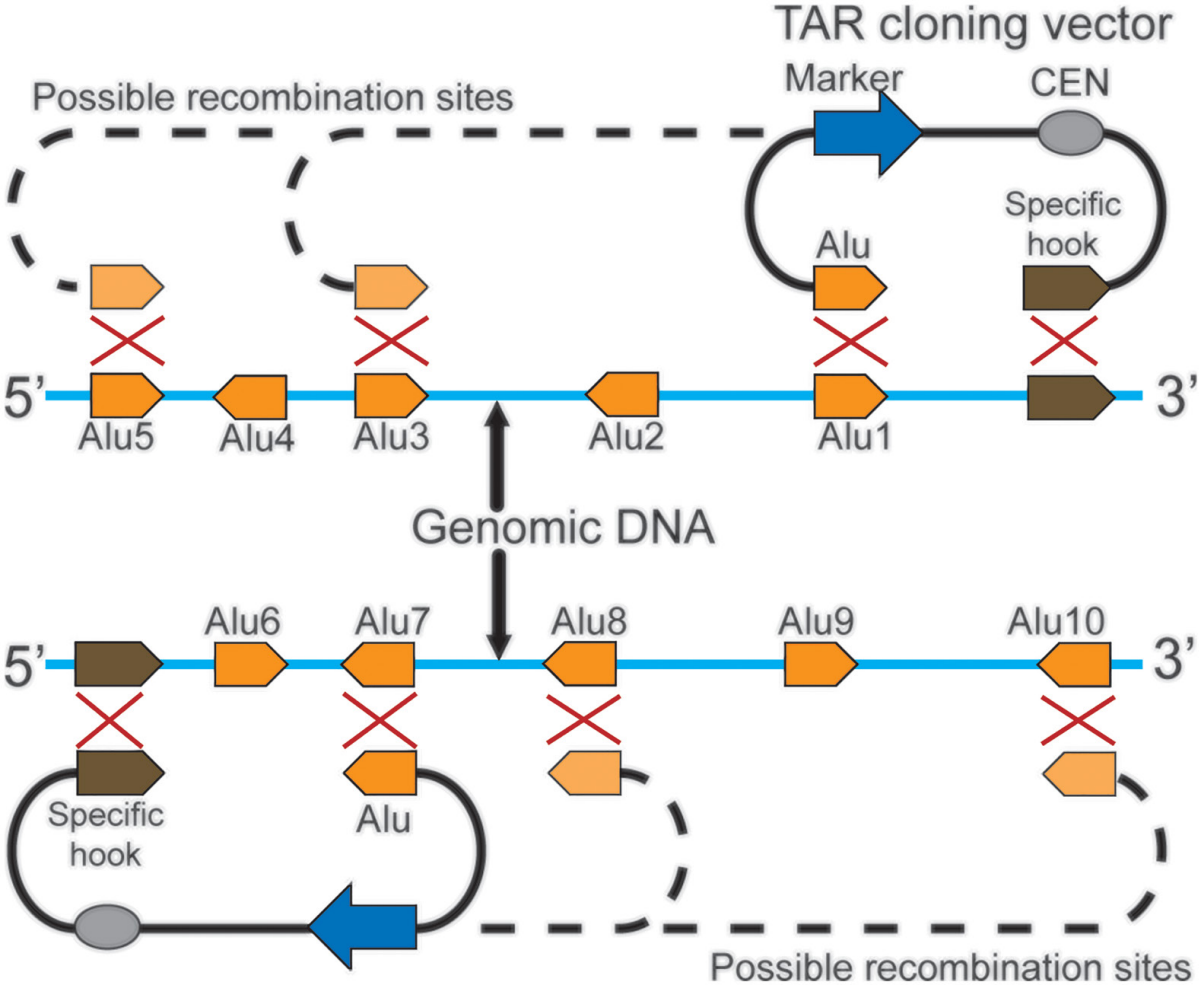
Isolation of a genomic region of choice by radial TAR cloning. The possible positions and orientations of the *Alu* sequences (in orange) in the genomic DNA are shown (from *Alu1* to *Alu10*). A unique hook in the TAR vector is marked in brown. TAR vector contains *CEN* (in grey) corresponds to the yeast chromosome VI centromere while a marker (in blue) is a selectable marker *HIS3*. Recombination during transformation between the hooks and genomic DNA leads to formation of the circular overlapping YAC molecules that extend from the unique hook to various *Alu* positions (*Alu1* to *Alu 5*). Changing an orientation of the specific hook in the TAR vector leads to cloning of the genomic DNA in the opposite orientation (*Alu 6* to *Alu 10*).

For many animal and plant genomes, only limited sequence information is still available. Therefore, TAR cloning with the vector containing two targeting hooks could be infeasible. To circumvent this limitation, another version of the TAR method was developed [7]. This version, branded as a radial TAR cloning, uses a vector as described in chapter 3.1 except that one specific hook has a unique sequence while another one has a common repeat sequence (i.e., *Alu* for the primate genomes or *B1* for the mouse genome) (Figure 4). Such a vector construction makes possible to isolate a region of choice as a set of nested overlapping fragments of different size that extend from the unique hook to the different recombination sites of a given repeat (*Alu1*, *Alu3* and *Alu5*) (Figure 4; above). By changing orientation of the unique targeting hook (from 3′ end to 5′ end), it becomes possible to isolate overlapping genomic regions that extend from the unique hook to recombination sites of a repeat located on the opposite side along the chromosome (*Alu7*, *Alu8* and *Alu10*) (Figure 4; below). The size of clones obtained by radial TAR cloning varies from 30 kb to 300 kb, reflecting the frequency and position of a repeat [7, 40–43]. It is worth noting that the yield of region-positive clones for radial TAR cloning is comparable to the yield obtained with the vector containing two targeting hooks. Radial TAR cloning has been applied to close the gaps in the human genome sequence and to isolate several specific regions from human and mouse genomes [7, 41, 43–46].

## APPLICATIONS OF TAR CLONING

### Functional genomics: isolation of full-size single-copy genes from complex genomes

Whereas cDNA clones are still widely used for gene expression [47–50], the full-size genes containing all exons, introns, and flanking regulatory elements become preferable because the scientific and especially biomedical communities show a keen interest in the mechanism regulating gene or gene cluster expression by means of alternative splicing, alternative promoter-enhancer usage, expression of non-coding RNAs from intronic regions, and 3D genome folding. TAR cloning of individual genes, containing all the necessary cis regulatory regions, provides a unique material for functional, structural (chapter 4.2), and population studies; for comparative genomics (chapter 4.3); long-range haplotyping (chapter 4.2); for biotechnology (chapter 4.4) and biomedicine (chapter 4.2). In addition, the TAR cloning technology may assist in designing diagnostics for genomic disorders caused by chromosomal rearrangements.

Over the past 25 years, TAR cloning has been used to isolate hundreds of full-size genes and gene clusters from genomes of humans, nonhuman primates, mice, and microbes [8, 20]. For example, functional analysis of several human genes, including 84 kb and 90 kb breast cancer genes *BRCA1* and *BRCA2* [6, 51], the 50 kb 3′ hypoxanthine phosphoribosyltransferase (*HPRT*) gene [7] that is mutated in Lesch-Nyhan syndrome, the 80 kb tumor suppressor gene *KAI1* [8], the 60 kb *NBS1* gene that is mutated in Nijmegen breakage syndrome, and the 30 kb *VHL* gene that is mutated in von Hippel–Lindau syndrome [8], demonstrated a high fidelity of the TAR-cloned genomic material. Accordingly, TAR-isolated genes were successfully used in transgenesis. In one example, a transgenic mouse carrying the entire 50 kb human *TERT* locus [52] was used to show that *in vivo* expression of human and mouse *TERT* genes differ significantly, raising awareness about the use of mouse models for human cancer and aging [53].

A substantial progress in gene functional studies was made upon combining the TAR-isolated full-size genes with the HAC-based gene delivery and expression vectors (chapter 4.7) [54, 55]. For example, the entire human *HPRT* locus was TAR-isolated as a 100 kb YAC/BAC clone [7], loaded into the HAC vector and then shown to complement the genetic defect of Hprt-deficient hamster CHO cells [56]. The examples of correction of genetic deficiencies in human patient-derived cells include the TAR-isolated genomic copies of *NBS1*, *BRCA1*, *VHL*, and *PKD1* genes loaded into the HAC that allows expression of the genes in target cells under conditions that recapitulate the physiological regulation of endogenous loci [57, 58]. More examples, indicating the accuracy of TAR cloning, include TAR-isolation and long-read sequencing of the 14 rDNA gene copies covering ~0.82 Mb of the human chromosome 21 rDNA cluster that enabled the accurate reconstruction of a high-quality 44,838 bp reference sequence [59]. TAR cloning and sequencing of the entire rDNA array end-to-end, including proximal and distal junction sequences, from the human chromosome 22 facilitated the reconstruction of the entire NOR (nucleolar organizer region) [60].

### Genetic basis of human diseases: separation of alleles and long-range molecular haplotyping

The word “haplotype” is derived from the word “haploid”, which describes cells with only one set of chromosomes, and from the word “genotype, which refers to the genetic markers of the organism. A haplotype is a group of genes within the organism that is inherited together from a single parent. Each haplotype has a frequency, which represents the proportion of chromosomes with the adjacent markers in the population. The haplotype frequency, a measure of the coordinated distribution of adjacent markers in the population, represents the correlation between those markers during inheritance.

In principle, the haplotypes or individual homologous chromosomes may be separated by pedigree analysis. However, this approach is laborious and restricted by the need to collect DNA samples from family members or different population groups. Other approaches such as microdissection of chromosomes or amplification of spermatocyte DNA are also time-consuming and labor-intensive making them unacceptable when analyzing many individuals. Modern computational methods do allow for haplotype analysis of samples of unrelated individuals [61–63]. However, all the approaches listed above have limitations, especially in resolving the phase of paternal and maternal chromosomes.

**Figure 5:**
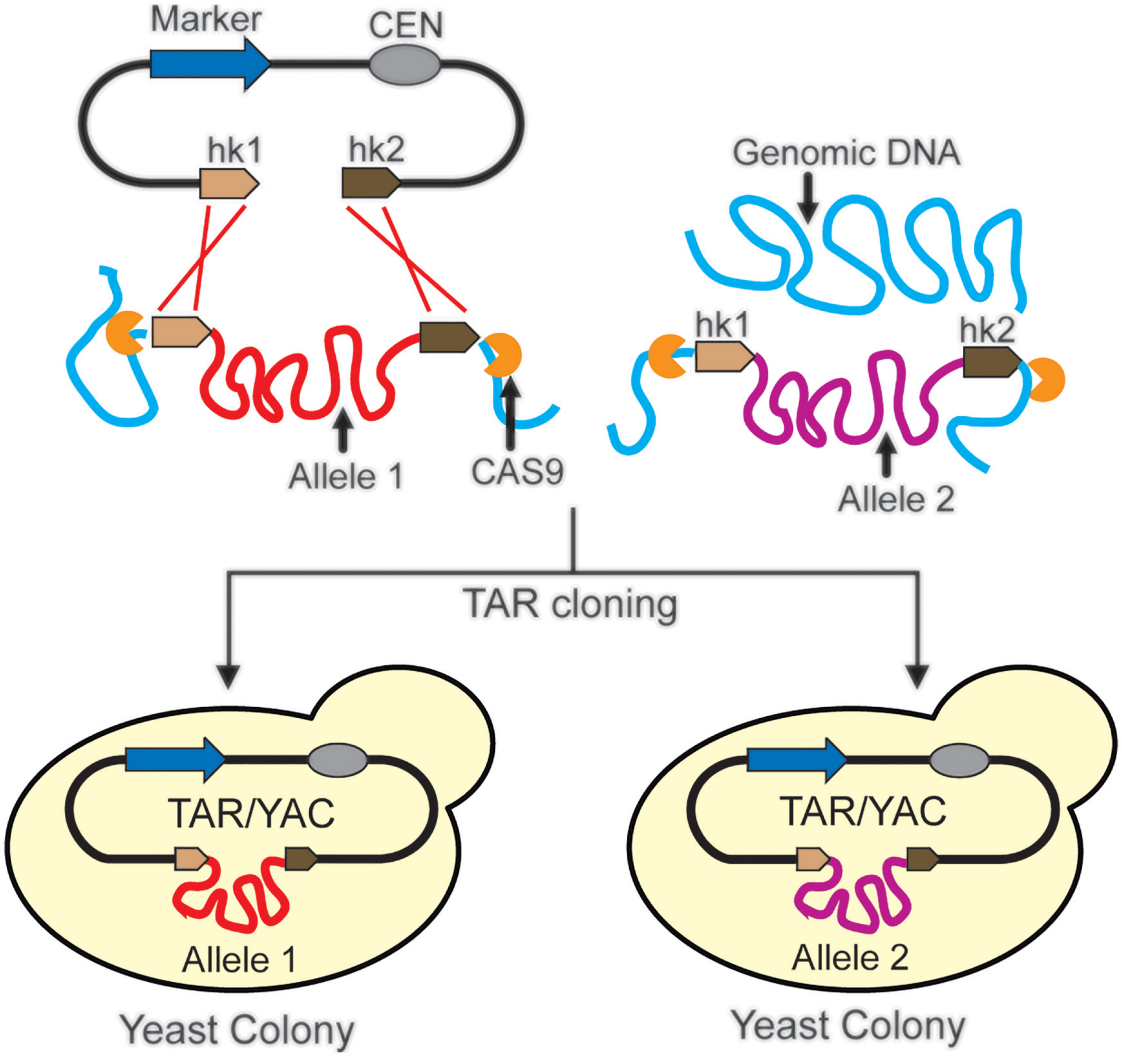
Separation of the alleles of the gene by TAR cloning. TAR vector contains a yeast selectable marker (in blue), a yeast centromere (*CEN*) and two unique hook sequences (hk1 in beige and hk2 in brown). TAR cloning results in multiple gene-positive transformants. Therefore, different alleles of a gene become separated into different yeast cells, allowing for further independent analysis.

TAR cloning represents a simple and reliable method used to resolve the haplotype characterization problem. Because recombination between the targeting hooks in the TAR vector and the homologous target sequences in the genome occurs at equal frequencies in both chromosomes, the parental alleles of a gene from multiple DNA samples can be simultaneously isolated in a single TAR cloning experiment (Figure 5). A representative example of application of TAR cloning for such a purpose is the separation of alleles of the 50 kb human *TERT* gene [64]. The *TERT* gene contains four VNTR (a variable number of tandem repeats) blocks, two of them located in intron 2 and two others in intron 6. To identify the parental *TERT* alleles, VNTR sequences in TAR isolates were examined and showed a specific allele-identifying combination of microsatellites at each of the polymorphic sites. Further sequencing of individual TAR isolates and analysis of segregation of these VNTRs in families revealed that all of them followed a Mendelian inheritance pattern [64]. Thus, TAR cloning allows separation of the haplotypes in individuals and has a potential to identify haplotypes that may contribute to disease(s).

**Figure 6:**
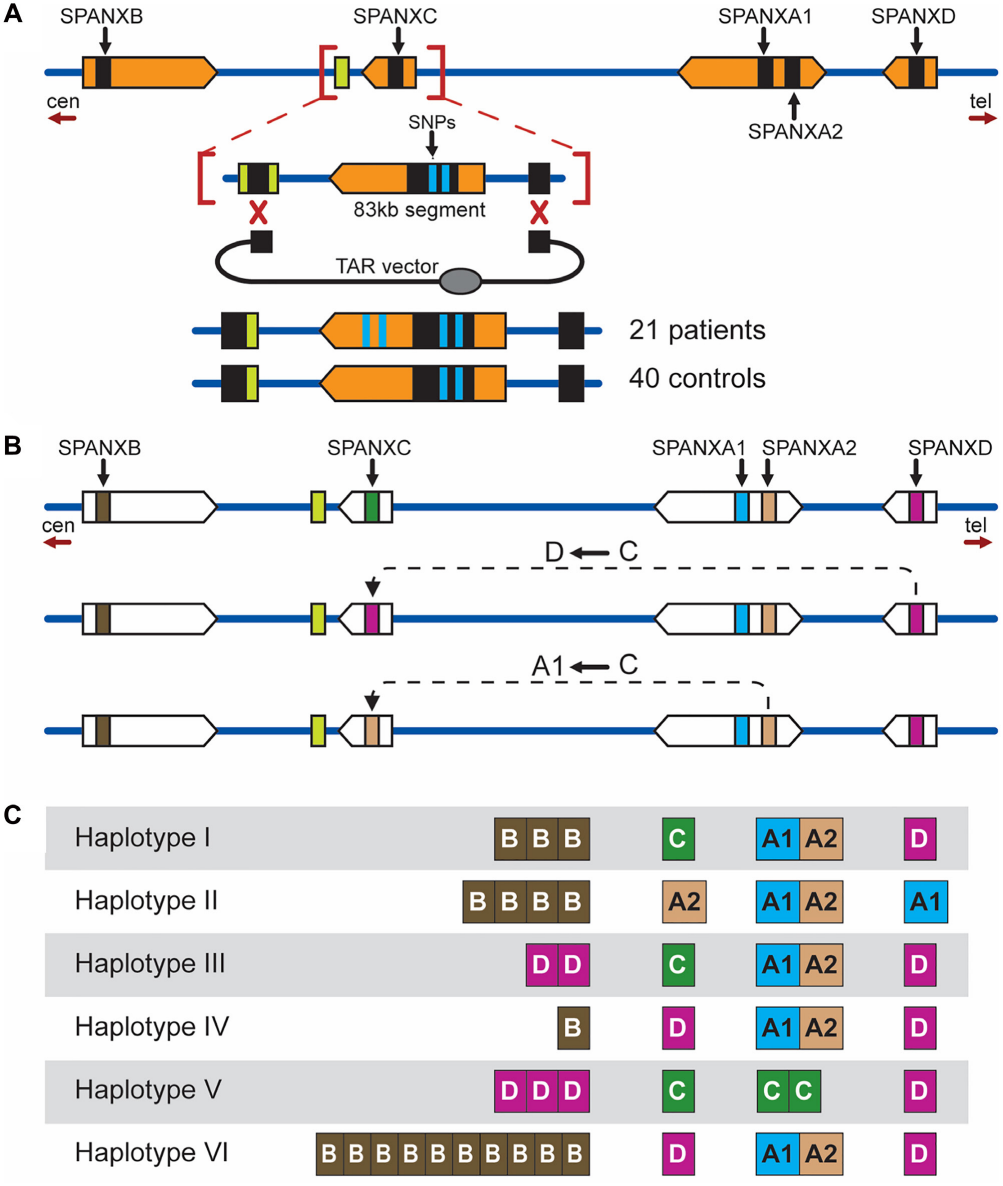
Long-range haplotyping of the Xq27 region in prostate cancer patients and healthy individuals. (**A**) TAR isolation of the *SPANXC* gene. The positions of *SPANX* genes are shown. The genes (in black) share 98% identity and reside within large segmental duplications (SDs) (in orange) with >95% identity. The *SPANXC* gene was TAR-isolated as an 83 kb segment from 21 patients and 40 healthy individuals in one TAR cloning experiment. The targeting hooks were chosen from the sequences outside the SD. The same strategy was used in mutational analysis of other gene family members. Orientation of the genes in the SDs towards the centromere (cen) and telomere (tel) is indicated: *SPANXD* to *SPANXC* and *SPANXA1* to *SPANXC*. (**B**) Gene conversion events between the SPANX-A/D gene family members (indicated by black arrows). (**C**) Six (I–VI) SPANX long-range haplotypes identified in the patients. Each column represents one of SPANX genes. For example, analysis of TAR isolates from individual VI revealed 10 copies of *SPANXB* at the *SPANXB* locus and conversion of *SPANXD* to *SPANXC* at the *SPANXC* locus.

More impressive that TAR cloning is suitable for large-scale analysis of long-range haplotypes in multiple, inherently heterozygous, individuals. An example of reconstructing long-range haplotypes in the cluster of the *SPANX*-*A/D* gene sub-family, located within a 750 kb region at Xq27-q28 that is presumable involved in the hereditary prostate cancer locus HPCX1 [65, 66], is TAR isolation of individual *SPANX* genes. The *SPANX-A/D* gene sub-family consists of five genes: *SPANX-C*, *SPANX-B*, *SPANX-A1*, *SPANX-A2*, and *SPANX-D* [67–69] (Figure 6A), with *SPANX-C* and *SPANX-D* genes separated by approximately 500 kb. Note that *SPANX-A/D* gene members have a level of homology close to 95% and reside within large segmental duplications (SDs) with >95% identity [70] (Figure 6A) that excludes a conventional PCR for gene mutational analysis. TAR cloning enabled the isolation of each member of this sub-family from dozens of normal individuals and patients during a quite short time [67–69] (Figure 6A). Further sequencing analysis of the TAR isolates revealed a high frequency of recombination between the genes due to gene conversion (for example, *SPANX-C* to *SPANX-A1* or *SPANX-C* to *SPANX-D*) (Figure 6B). As seen, the corresponding recombinational interaction operates over a long distance (~500 kb) [71]. Sequencing data allowed to reconstruct long-range *SPANX* haplotypes [68] (see examples in Figure 6C). Moreover, sequence analysis and long-range haplotyping in normal individuals and patients revealed no disease-specific mutations or genomic alterations within the *SPANX* gene cluster that excluded a 750-kb region at Xq27-q28 as a candidate locus for prostate malignancy [69].

To summarize, TAR cloning is a unique tool to rapidly and accurately isolate both alleles of a gene and build long-range haplotypes for multiple heterozygous individuals. In addition, TAR cloning can help to develop diagnostics for disorders caused by genomic rearrangements that would provide the important foundation for the biomedicine of the future.

### Comparative genomics and evolutionary studies: isolation of gene homologues

As described in chapter 2.2, 15% divergence between the targeting hook sequences in the TAR vector and target 5′ and 3′ ends of the genomic region does not prevent recombination allowing TAR isolation of homologous regions from different species [21]. For example, the efficiency of cloning of the mouse *HPRT* gene using TAR vector containing the human targeting hooks having 14% divergence with the 5′ and 3′ ends of the mouse *HPRT* gene was the same as cloning of the human *HPRT* gene [8]. Other examples of application of TAR technology of the large genomic regions from evolutionary close species include: the *BRCA1* tumor suppressor gene [72], the microcephaly gene *ASPM* controlling brain size [73], the *SPANX* gene sub-family [70], and the *NBS1* and *ATM* genes involved in DNA repair [20]. These genes were isolated from human and non-human primate species using TAR vectors containing the human targeting hooks corresponding to 5′ and 3′ gene-flanking regions with the hook sequences being 14–15% diverged. The subsequent sequence analysis then enabled the reconstruction of the evolutionary history of these genes [67, 72, 73].

Sequence analysis of the *ASPM* gene, which encodes for a mitotic spindle protein [74], revealed a high conservation in both coding and noncoding regions and allowed to infer that evolution of this gene was under positive selection in hominoids [73]. That study also suggested that the evolutionary selection of *ASPM* in the African hominoid clade preceded hominid brain expansion by several million years and strongly correlated with differences in cerebral cortical size [73].

The *BRCA1* gene is involved in many cellular functions, including DNA replication, cell-cycle checkpoint activation, gene transcriptional regulation, DNA damage repair, kinetochore function, and centrosome function. Analysis of the synonymous versus non-synonymous substitution ratio in the coding region of *BRCA1* revealed that the coding (internal) sequence has evolved under positive selection while the terminal regions of *BRCA1*, which encode the BRCT domain and RING finger, are almost identical in all primates [72]. Interestingly, the human *BRCA1* gene contains 129 *Alu* elements, accounting for ~42% of the entire gene sequence. It was shown that a significant fraction of germline *BRCA1* mutations in hereditary breast and ovarian cancers are deletions and duplications caused by homologous *Alu-Alu* recombination [75], resulting in gene inactivation [76]. Sequence analysis of TAR-isolated *BRCA1* homologues revealed that the *Alu* repeats involved in disease-associated genomic rearrangements are conserved in nonhuman primates, suggesting their functional significance. Additionally, *Alu*-mediated rearrangements, including *Alu*-associated deletions and *Alu* transpositions, are the major force of evolutionary changes in noncoding *BRCA1* sequences [72].

**Figure 7:**
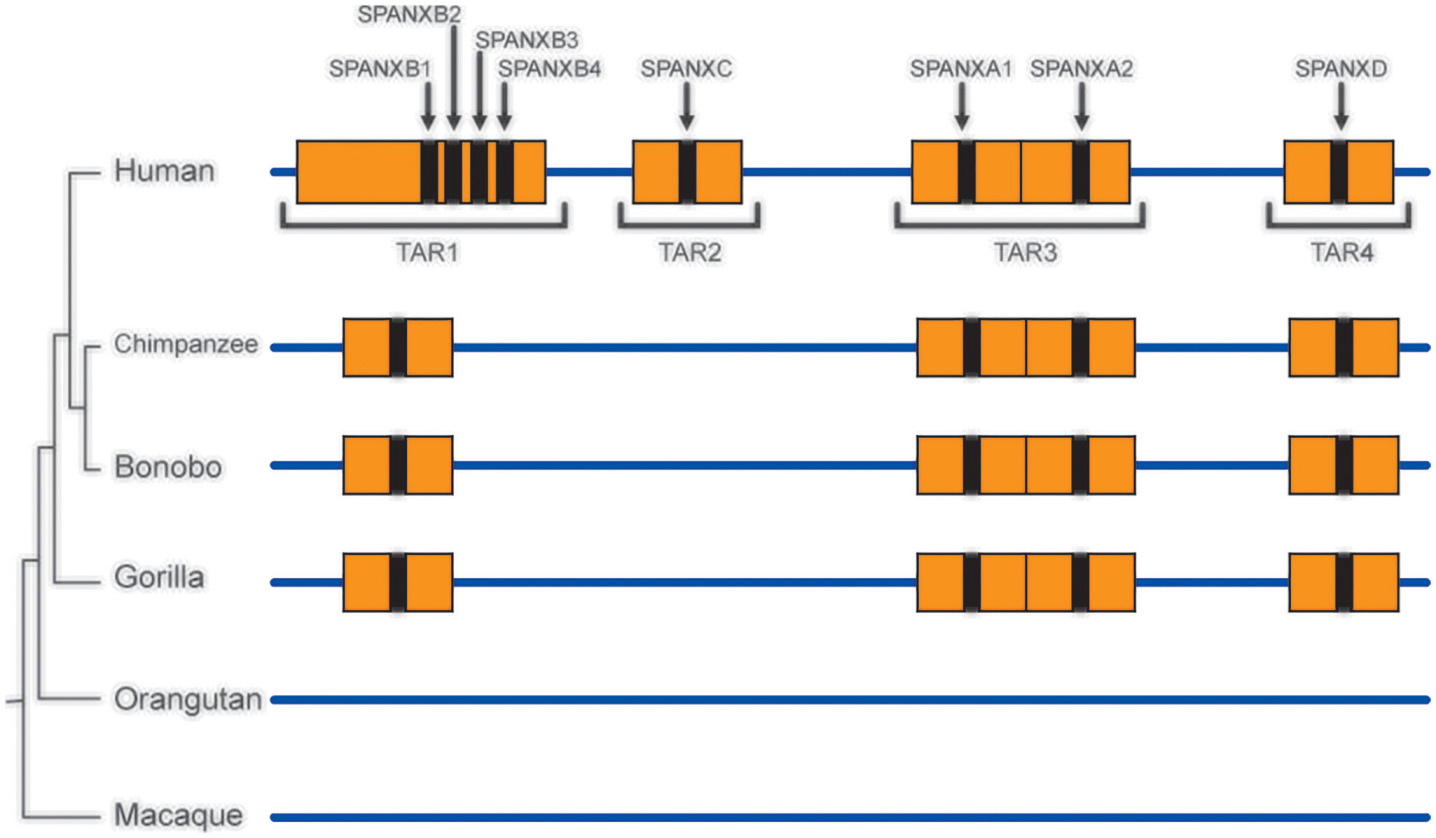
TAR cloning of the *SPANX-A/D* genes and their organization in primates. Syntenic genomic fragments containing different members of the *SPANX-A/D* gene family from the human, chimpanzee, bonobo, gorilla, orangutan, and macaque were TAR-isolated. Unique targeting hooks in the TAR vectors were chosen from the human genome regions flanking the SDs where the *SPANX-A/D* genes are located. Note that the divergence of these regions between primates is approximately 15%. Sequencing of the TAR isolates (TAR2) from chimpanzee, bonobo, gorilla, orangutan, and macaque showed that these primates do not contain the *SPANX-C* gene along with the SDs. Sequencing of the TAR isolates (TAR1) from primates showed that the *SPANXB* gene along with the SDs is absent in the orangutan and macaque. In human, the *SPANXB* gene is amplified from one up to 14 copies that is human specific.

The most unexpected example is reconstruction of the evolutionary history of the *SPANX-A/D* gene sub-family in primates [70, 71]. As described above (see chapter 4.2), these genes are located within 95% identical SDs. The latter precludes the detection of lineage-specific amplification of these genes by routine PCR or next generation sequencing analyses of syntenic chromosomal segments. TAR cloning enabled to overcome that problem, and the syntenic fragments from human, chimpanzee, bonobo, gorilla, orangutan, and macaque were isolated and sequenced. Remarkably, the corresponding TAR clones from syntenic regions of chimpanzee, bonobo, and gorilla genomes did not contain the *SPANX-C* gene that means that this gene is human-specific [70] (Figure 7). Analysis of the *SPANX-B* containing duplication revealed a variable number of a 12 kb tandem repeat carrying *SPANX-B* within SD, ranging from 1 to 14 copies, that is present only in humans [71]. More interesting, further analysis of the TAR isolates revealed that the *SPANX-A/D* gene sub-family is absent in orangutan and macaque (Figure 7), making this gene sub-family specific for the human lineage.

To summarize, despite the progress in genome sequencing, a quick, an efficient and a simultaneous TAR isolation of gene homologues from different species provides an opportunity to address fundamental questions in environmental evolution.

### Biotechnology: selective isolation and assembly of biosynthetic gene clusters (BGCs) from individual microbial genomes and environmental DNA samples

NPs BGCs and their derivatives are the major source of pharmacological agents and industrial compounds [77]. With ineffectiveness of most antibiotics and the spread of drug-resistant pathogens, the discovery of new BGCs and developing them into drugs has become an urgent necessity. So, it is not surprising that TAR cloning became a widely used, an effective, rapid, and accurate tool for capture of BGCs from microbial genomes and for their cloning and assembly from collections of overlapping environmental eDNA clones (summarized in [78, 79]).

**Figure 8:**
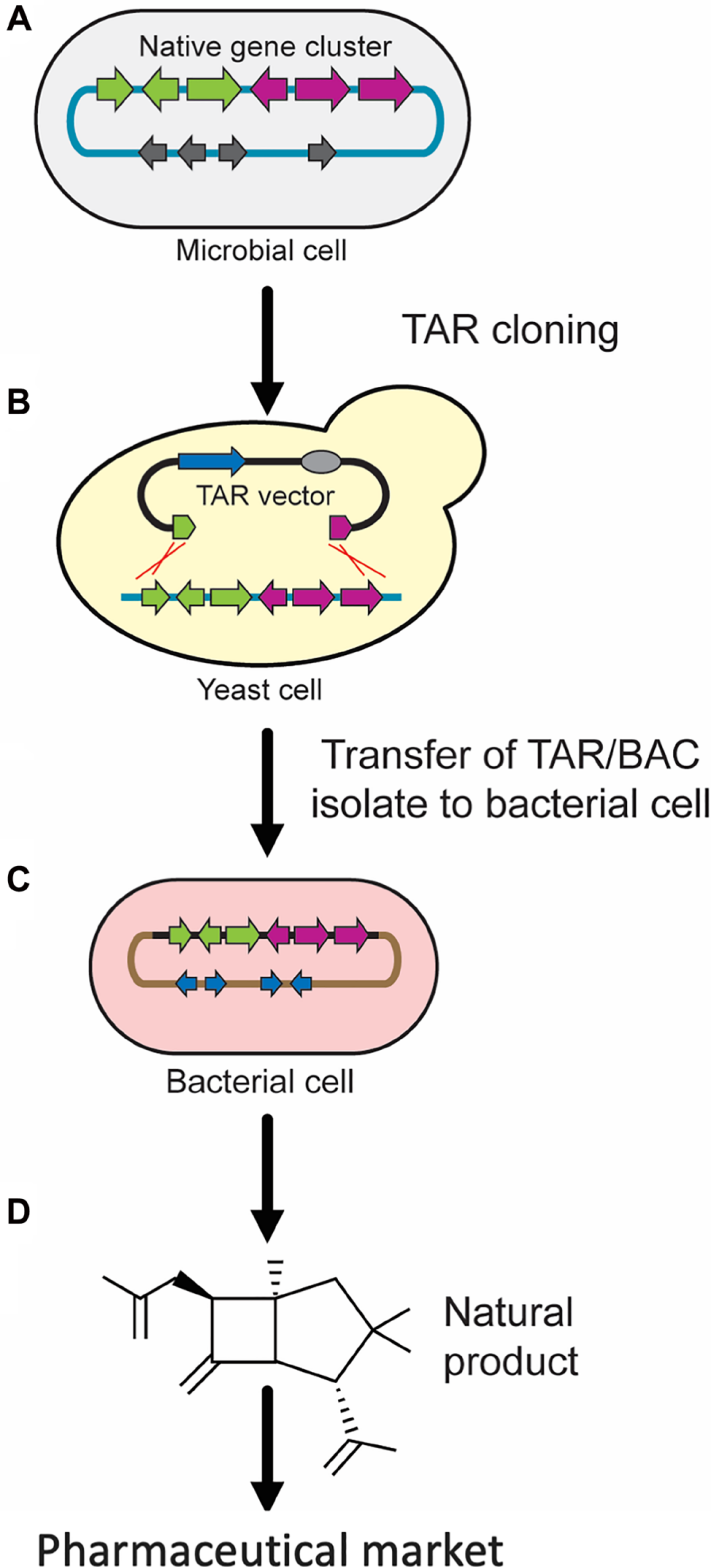
TAR cloning of natural product biosynthetic gene clusters (BGCs) from microbial genomes. (**A**) Organization of a native gene cluster located on a chromosome propagated in a microbial cell. (**B**) Isolation of BGC by TAR vector where the hooks (in green and in purple) correspond to the ends of the gene cluster. TAR is based on homologous recombination between the hooks of TAR vector and co-transformed genomic DNA isolated from host microbial cells that leads to formation of a circular YAC/BAC construct. In the TAR vector, the hooks or homology arms (in green and in purple) correspond to the ends of the cluster. (**C**, **D**) TAR-cloned gene cluster is transferred to bacterial cells and, if necessary, integrated into the chromosome of a host strain for basic research or production of natural compounds.

Over the past decade, there are many successful examples of TAR cloning of BGCs from bacteria and collections of soil-derived eDNA clones for commercial purposes as well as for functional studies [11, 13, 78, 80–104]. In many cases, cloning of BGCs from cultured microorganisms is possible using the protocol with TAR vector lacking an *ARS* (chapter 3.1) (Figure 2) [105]. However, a lot of microbial genomes are low in the density of *ARS*-elements and therefore it is highly likely that some BGCs do not possess *ARS*-like sequences. For such cases, a TAR cloning protocol, that uses a counter-selectable marker, adapted for genomes that have few or no *ARS*-like sequences is preferrable (chapter 3.2) (Figure 3) [12]. Figure 8 describes a general scheme of TAR isolation of a natural product biosynthetic gene cluster from a microbe with its following transfer to a bacterial host strain for production of the natural compound or basic research.

One of the examples of TAR capture of BGCs from eDNA samples is screening of eDNA megalibraries [106]. Isolation and further structure elucidation of metabolites obtained through heterologous expression of these gene clusters identified three new fluostatins (F, G, H) that had not been characterized before from studies of cultured species. Two other groups described TAR capture of overlapping soil-derived eDNA clones followed by their re-assembly into ~90 kb BGCs [85, 87]. TAR capture of BGC to yield a new antibiotic was described by Yamanaka et al. [96]. More specifically, a nonribosomal peptide synthetase cluster 73 kb in size was isolated from the marine actinomycete *Saccharomonospora sp*. CNQ-490 to produce lipopeptide antibiotic taromycin A in the model expression host *Streptomyces coelicolor* [96]. Later TAR cloning of the 6-demethylchlortetracycline BGC from *Streptomyces* *aureofaciens* was described [107]. A similar approach was used to isolate the 54 kb aromatic polyketide antitumor agent cosmomycin BGC from *Streptomyces* bacteria [108] and the putative thioviridamide-like gene cluster, including up and downstream flanking regions, from *Streptomyces sp*. NRRL S-4 [109]. TAR cloning also allowed discovery and isolation of the 67 kb malacidins as calcium-dependent antibiotics with activity against multidrug-resistant Gram-positive pathogens [110]. In another work, a 33 kb genomic region that includes a cryptic antibiotic biosynthesis gene locus was identified and TAR-captured from human pathogenic *Nocardia* strain and then expressed in the *Streptomyces* host revealing it to be a source of the brasiliquinones and benz(a)anthraquinone antibiotics [111].

More recent examples of TAR application is capture of large BGCs with high G+C content, including 98 kb tylosin (tyl), 128 kb daptomycin (dpt), and 127 kb salinomycin (sal) with their further heterologous expression in *Streptomyces coelicolor* M1146 to produce tylosins in the resulting recombinant strains [104 ] and 127 kb stictamycin (sal), an aromatic polyketide antibiotic isolated from a New Zealand Lichen-Sourced *Streptomyces* species with activity against *Staphylococcus aureus* that is the most pathogenic (it is typically causes skin infections and sometimes pneumonia, endocarditis, and osteomyelitis) [112]. Another group reported a yeast-based platform that exploits TAR cloning for capture, expression, and analysis of a BGC encoding a nonribosomal peptide eponemycin, a novel antibiotic, and TAR capture of TMC-86A that belongs to a family of peptide natural products to clarify the biosynthesis of these important proteasome inhibitors [113].

Recently Awal and co-authors [114] have performed an extraordinary work on isolation of 30 specific genes, that are comprised within the compact magnetosome gene cluster (MGCs), from the *Magnetospirillum* bacteria using the TAR cloning technique. In species of *Magnetospirillum*, biosynthesis of magnetosomes is a complex process, governed by these 30 genes. A further reconstruction, transfer, and analysis of this entire magnetosome cluster is promising for engineering the biomineralization of magnetite crystals with different morphologies that would be valuable for biotechnical applications [114].

Another notable example was demonstrated by Santos-Aberturas and co-authors [115]. Thioviridamide is a structurally novel ribosomally synthesized and post-translational modified peptide (RiPP) produced by *Streptomyces olivoviridis* NA005001. This peptide is characterized by a series of thioamide groups and possesses potent antiproliferative activity in cancer cell lines. The authors investigated the diversity of thioviridamide-like pathways across sequenced bacterial genomes and three diverse members of this family were TAR-captured from the genetically intractable *Streptomyces sclerotialus* bacterial strain [115].

CRISPR/Cas9-mediated TAR cloning (chapter 3.1) was applied to isolate the core genes for plipastatin biosynthesis from *B. amyloliquefaciens* HYM12 followed by their highly efficient expression in a heterologous system of *Bacillus subtilis* [116]. The same strategy was applied for isolation of staurosporine BGC 22.5 kb in size from the native producer and then introduction into heterologous hosts *Streptomyces avermitilis* [117]. Staurosporine is the most well-known member of the indolocarbazole alkaloid family. It can induce apoptosis of many types of cells as a strong protein kinase inhibitor and is used as an important compound for the synthesis of the antitumor drug [117].

A recent example of reconstruction of BGC is TAR cloning and further reassembly of the gene cluster of microcystin-LR from *Microcystis aeruginosa*, a species of freshwater cyanobacteria that can form harmful blooms of economic and ecological importance [118]. *Microcystis aeruginosa* produces microcystin-LR (MC-LR), the most common cyanotoxin. Isolation of this cluster allowed to study the biosynthetic pathways and molecular mechanisms of MC-LR.

To summarize, TAR method provides a powerful, effective, and accurate tool for isolation of natural product biosynthetic gene clusters for biomedicine, biotechnology, and fundamental research.

### Biomedicine: assembly and cloning of synthetic viruses and bacteriophages

**Figure 9:**
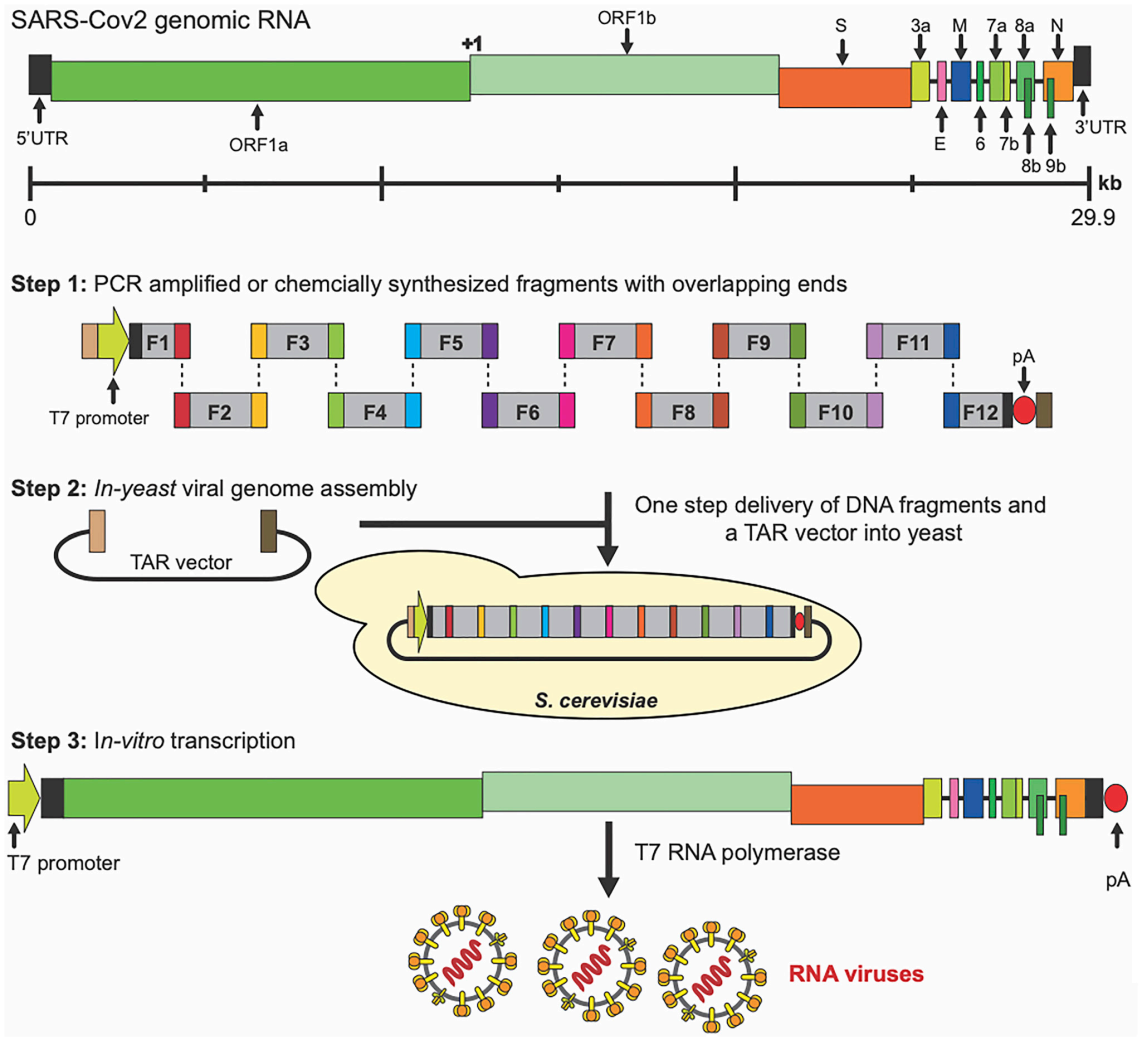
One-step SARS-COv2 genome assembly in yeast *S. cerevisiae* using transformation-associated recombination (TAR) in yeast [120]. **Step 1:** Genomic DNA of SARS-CoV-2 was divided into 12 overlapping fragments. F1 fragment was fused with T7 promoter at its 5′ terminus (in green and in light brown rectangle) and F12 fragment was fused with poly(A) (pA) at its 3′ terminus (in dark brown). TAR vector has targeting hooks (in light brown and in dark brown) having homology to T7 promoter (the light brown part) and the sequence fused with F12 (in dark brown). **Step 2:** One-step delivery of 12 fragments and the linearized TAR vector into yeast cells and following assembly of the viral full-length cDNA by homologous recombination. **Step 3:** The EagI site at the 5′ end of the fragment F12 was cleaved to linearize the molecule. The linearized molecule was used as the template to synthesize the viral infectious full-length RNA of SARS-Cov2 using T7 RNA polymerase.

TAR cloning is used to genetically engineer synthetic viruses with novel properties to design a new generation of vaccines. Recently Kurhade and co-authors summarized the status of TAR-assembled viral genomes, including SARS-CoV-2, and their further applications for studying the pathogenesis and replication of viruses and the development of vaccines [119]. Figure 9 shows an example of construction of a synthetic genomic RNA for the respiratory syndrome coronavirus 2 (SARS-Cov2) [120]. Step 1 includes PCR amplification or chemically synthesized 12 small viral fragments having overlapping ends. F1 fragment is fused with T7 promoter at its 5′ terminus. F12 fragment is fused with polyA (pA) at its 3′ terminus. Step 2 describes viral genome assembly in yeast as YAC molecules containing the full-length viral DNA using TAR vector containing two targeting hooks with the homology to the 5′ and 3′ ends of the PCR-amplified F1 and F12 fragments. Step 3 incudes *in vitro* transcription of the assembled genome with T7 RNA polymerase to generate the infectious full-length viral genomic RNA.

Using a similar TAR-based approach, two other groups reconstructed RNA viruses, including members of the Coronaviridae, Flaviviridae, and Pneumoviridae families [29, 30]. They used sub-genomic fragments of diverse origins: viral isolates, cloned viral DNA, clinical viral samples, or synthetic DNA fragments. These fragments were reassembled as YAC molecules in one step in yeast using transformation-associated recombination. Then using T7 RNA polymerase infectious virus RNAs were generated for the rescue of a viable viruses. The same group also engineered the SARS-CoV-2 virus using chemically synthesized synthetic DNA fragments of the virus. Only a week was required to generate the full-length viral genomic RNA [29, 30].

Another impressive example of TAR-based assembly of the entire viral genome is reconstruction of infectious laryngotracheitis virus (ILTV), known as Gallid alphaherpesvirus-1 [121]. In this case, the authors generated overlapping cosmid clones, that encompassed 90% of the 151 kb ILTV genome. Homologous recombination between the clones in yeast allowed to develop the full-length genome of the ILTV virus [121]. TAR-based approach allowed to manipulate the constructs by modifying the genes encoding virulence factors that facilitated the development of the improved virus vaccines and establishing ILTV-based viral vectors for expressing immunogens of other avian pathogens.

Recently the TAR technology allowed to rescue different strains of feline infectious peritonitis virus without multiple cloning steps [122]. That virus causes a deadly disease in cats for which there is no effective vaccine. In this study, the authors provided an improved TAR-based system and constructed infective cDNA in one week. This allowed them to construct an infectious virus that would benefit for the vaccine development and pathogenic mechanism research [122]. Similarly, a combination of PCR and TAR cloning allowed to construct the genome of the *Autographa californica* multiple nucleopolyhedrovirus (AcMNPV) [123]. TAR cloning was used to assemble the overlapping fragments into a complete herpes simplex virus type 1 genome (HSV-1) [124] and has been also adapted to directly clone the genome of large human cytomegalovirus (HCMV) [125].

Bacteriophages, also known as phages, are viruses that infect and replicate only in bacterial cells. They are ubiquitous in the environment and are recognized as the most abundant type of organisms on earth. Recently they have received renewed attention for their potential to address the rise of multidrug-resistant bacteria resulting from the overuse of antibiotics [126, 127]. Therefore, modification of phages by homologous recombination in yeast or their assembly by TAR cloning provides a promising approach against antibiotic-resistant bacteria. Though the phages have a limited range of hosts that hinder their effectiveness, TAR engineering may expand the bacterial host range, and improve phage pharmacological efficacy. One among multiple examples of TAR engineering of phages is a full phiX174 genome assembly with the yield 44% of the required clones [128]. More examples of TAR-based assembly of phage DNA fragments into complete genomes in yeast, followed by transformation into the hosts to produce activated phages for drug production are summarized by Jia et al. [127].

### Synthetic biology: assembly of microbe genomes and Mb-scale human DNA fragments

**Figure 10:**
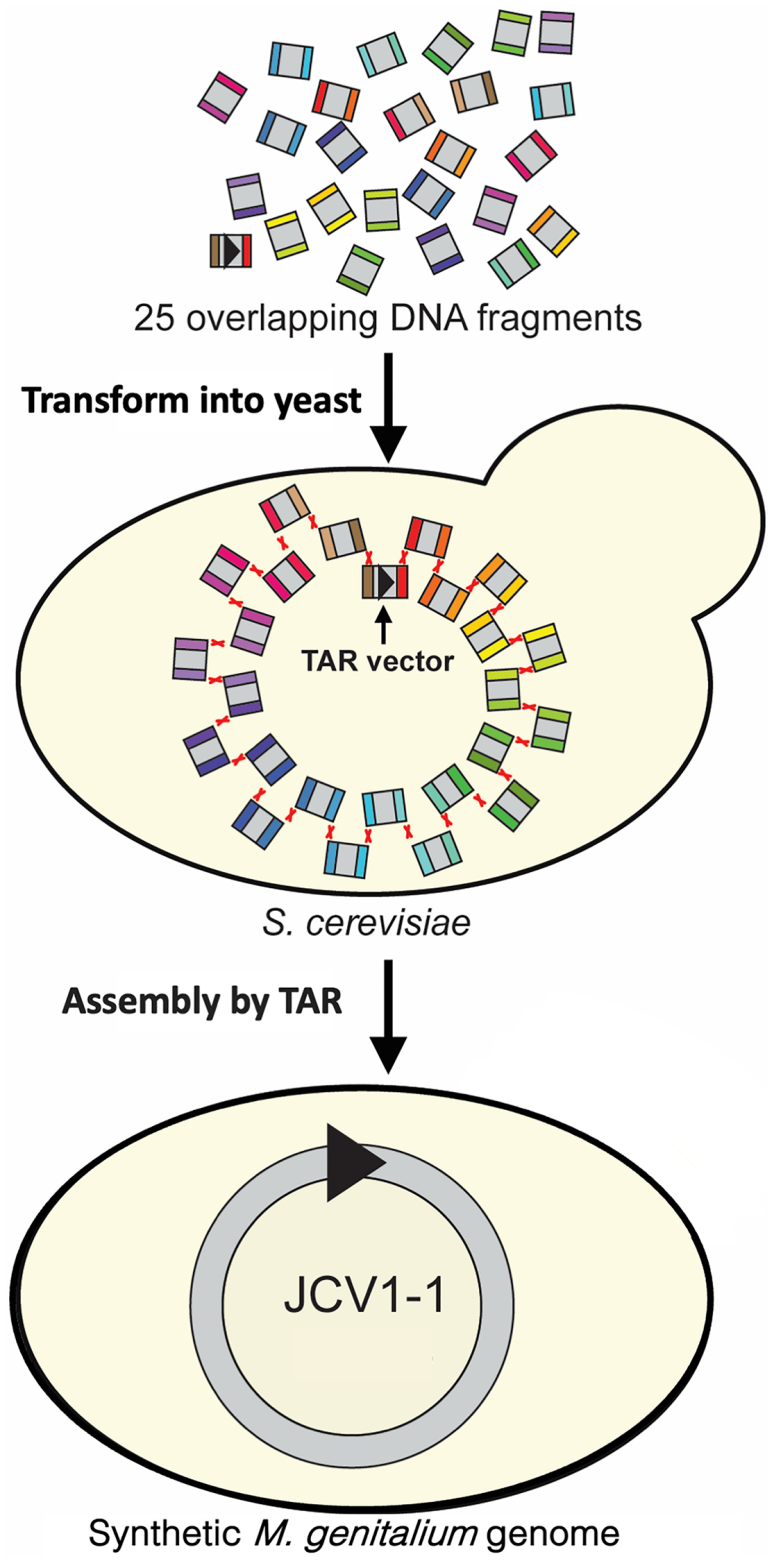
Assembly of a synthetic *Mycoplasma genitalium* genome from 25 overlapping DNA fragments in the *Saccharomyces cerevisiae* yeast cell [129]. DNA fragments from 17 kb up to 35 kb each along with the TAR vector were transformed into yeast cell. Homologous recombination between the ends of the fragments led to one-step assembly of the synthetic *M. genitalium* genome. This assembled genome called JCV1-1, 590,011 bp in size, includes the TAR vector sequence (marked by black triangle). TAR vector has targeting hooks having homology to the first and the last fragments of *M. genitalium* genome.

An era of assembly of synthetic microbial genomics began in 2008. A team of the J Grag Venter Institute described the first synthesized and assembled bacterial *Mycoplasma genitalium* genome (JCV1-1) approximately 590 kb in size [82]. The *M. genitalium* genome was assembled using a combination of *in vitro* enzymatic and *in vivo* TAR cloning approaches. First, 25 DNA fragments with an average length of 24 kb were assembled *in vitro* into four 144 kb fragments having the homological ends to each other. Then TAR method was applied to assemble the whole *M. genitalium* genome. Later, using the recombination machinery in yeast, this team assembled the 25 overlapping DNA fragments in yeast into a complete microbial genome JCV1-1 ~590 kb in size in a single step (Figure 10) [129].

For the past decade TAR cloning promoted a significant progress in synthetic biology. Another example of TAR application is assembly of eleven ~100 kb overlapping DNA fragments into the complete 1.1 Mb *M. mycoides* genome that was then transferred into closely related *M.* *capricolum* cells to form chimeric *M. mycoides* cells. The novel chimeric cells were capable of self-replication and revealed the expected phenotypes [83]. At present, TAR cloning is routinely used to assemble the whole genomes from either synthetic or natural DNA molecules; for example, 0.8 Mb *M. pneumoniae* [25], *1.5 Mb Acholeplasma laidlawii* [26], and 1.6 Mb *Prochlorococcus marinus MED4* genomes [27].

The potential of TAR cloning in yeast as a universal host for *in vivo* assembly of large eukaryotic chromosomes has been also demonstrated [130]. One of the examples describes assembly of two eukaryotic chromosomes, each ~500 kb in size, of the algal *Phaeodactylum tricornutum* genome (27.4 Mb) [28]. The TAR strategy has been also applied to assembly four-, five-, and six-gene complex pathways to generate yeast cells synthesizing beta-carotene, and violacein [131] and to engineer complex pathways, such as the synthesis of amorphadiene and vanillin [132, 133]. Thus, TAR cloning is adapted as a reliable and accurate method for the assembly of synthetic genomes and pathways [134, 135].

### Synthetic biology: construction of human artificial chromosomes with a defined structure

For the past two decades, HAC-based vectors have been widely used for gene delivery, new anticancer drug screening, discovery of novel genes involved in chromosome transmission, and for the study of centromere assembly and function [54, 55, 136, 137]. The HACs have a potential for gene therapy, and regenerative medicine [138]. The HACs are maintained stably as an additional 47^th^ chromosome in human cells over multiple generations due to the presence of functional kinetochore [137]. Because the HACs can carry the genes with all regulatory elements, this allows the genes to mimic the pattern of the natural gene expression.

**Figure 11:**
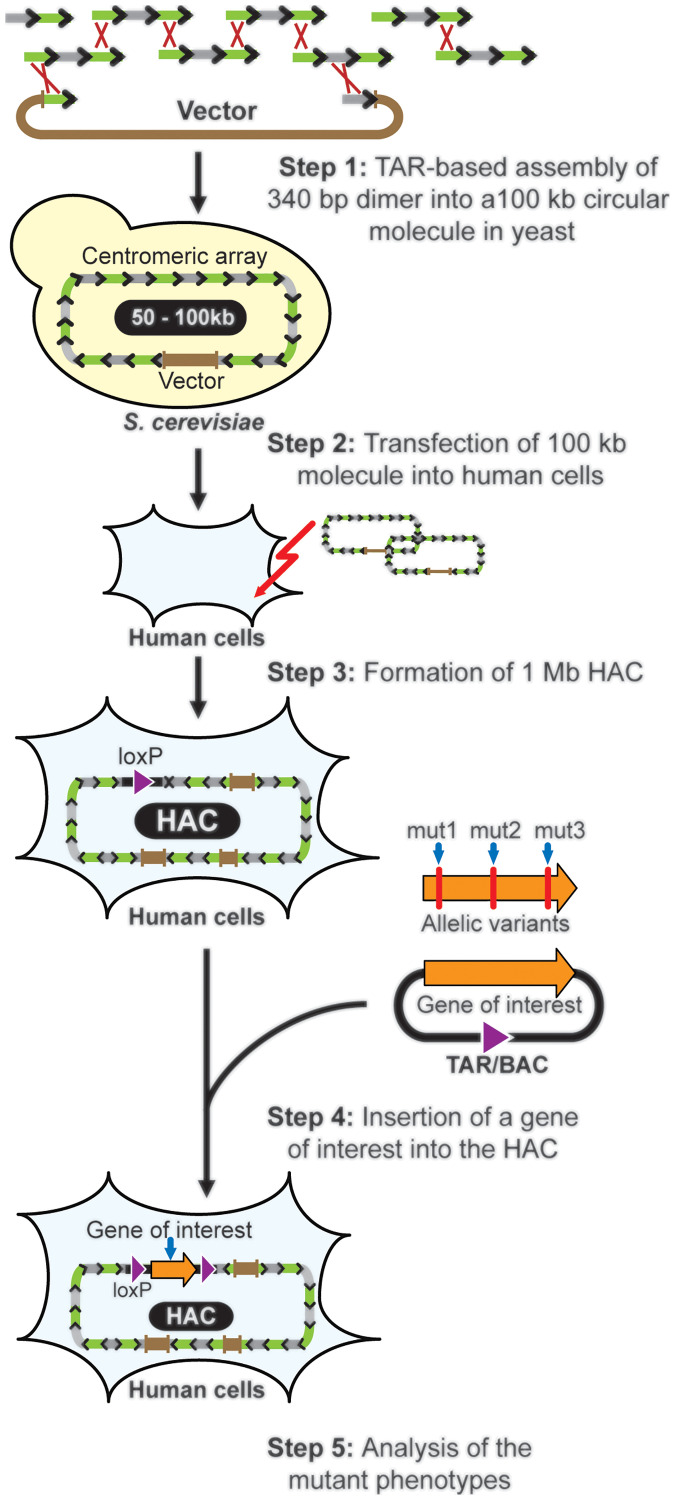
HAC-based gene delivery vector formation. **Step 1:** Assembly of the 340 bp DNA dimers into 50–100 kb centromeric arrays by TAR cloning in yeast *S. cerevisiae* cells. TAR vector contains yeast and bacterial cassettes, a mammalian selectable marker (the Blasticidin resistance gene), and the 340 bp dimers as the targeting hooks. TAR vector linearized between the hooks and a mixture of dimers are transformed into yeast cells. End-to-end recombination of the DNA dimers followed by interaction of the recombined fragments with the vector hooks results in formation of the 50–100 kb centromeric arrays as circular YAC/BAC molecules. **Step 2:** TAR vector contains a mammalian selectable marker (the Blasticidin resistance gene) that allows a TAR/BAC clone containing a 100 kb centromeric array to be transferred to and maintained in human cells. **Step 3:** Formation of the HAC accompanied by input alphoid DNA multimerization up to ~1 Mb [141]. **Step 4:** A TAR-isolated gene of choice or an allelic variant of a gene (mut1, mut2 or mut3) is loaded into the single gene acceptor loxP site of the HAC by Cre-loxP mediated recombination. **Step 5:** Analysis of the mutant phenotypes of the gene.

In 2005 Ebersole and co-authors described a method to construct synthetic alphoid DNA arrays with a predetermined structure [139]. Using *transformation-associated* recombination in yeast, it was shown that 2mer or 4mer or 5mer alphoid DNA repeats consisting of alphoid 170 bp monomers and having the ends homologous to each other may be one-step TAR-assembled into long synthetic alphoid DNA arrays varying in size from 50 to 140 kb (Step 1: Figure 11). After transfection of such arrays into human cells, *de novo* HACs are generated, ranging in size from 1 to 10 Mb due to amplification of the input alphoid DNA arrays (Steps 2 and 3) [139–143]. Because any nucleotide in the original dimer can be easily changed before its amplification, this TAR-based method allows to identify the critical regions of the alphoid repeat for *de novo* centromere seeding.

A decade ago, a HAC termed as the alphoid^tetO^-HAC was constructed, using the TAR-based method. The first step included one-step assembly in yeast of 34 overlapping 348 bp in size alphoid DNA dimers to form a 120 kb synthetic array. In each dimer, one monomer contained a tetO sequence in place of the CENP-B box that allowed these dimers to be targeted specifically with tetR-fusion proteins. The 120 kb array was transformed into human fibrosarcoma HT1080 cells forming a 1.1 Mb HAC [141, 144]. Further analysis of the HAC revealed that heterochromatin is incompatible with centromere function and that centromeric transcription is important for centromere assembly and maintenance [55, 137, 144]. The alphoid^tetO^-HAC was adapted for gene delivery and expression studies that allowed the TAR-isolated genomic copies of the genes (*HPRT*, *VHL*, *NBS1*, *BRCA1*, *PDK1*, *ATM*, rDNA) to be inserted into a unique gene loading LoxP site for further analysis of allelic variants [56–58, 145, 146] (Steps 4 and 5). Importantly, the complementation of mutant phenotypes arising from stable gene expression can be reversed by inactivating HAC’s kinetochore in proliferating cell populations, a feature that provides a control for phenotypic changes attributed to expression of HAC-encoded genes [57, 58, 146].

To conclude, the TAR-based method has a general application in elucidating the role of other tandem repeats in chromosome organization and dynamics. It is worth noting that in 2008 Gibson and co-authors applied a similar strategy to assemble a complete synthetic *Mycoplasma* genome from 25 overlapping DNA fragments [129].

## CONCLUSIONS

TAR cloning has become a valuable procedure for the selective and efficient isolation and manipulation of large DNA molecules. Its ability to isolate unperturbed native genomic regions provides a basis for a multitude of practical applications in biomedicine and biotechnology. The availability of full-length genes containing exons and introns with 5′ upstream and 3′ downstream regulatory sequences will catalyze major breakthroughs in functional, structural, and comparative genomics, diagnostics, gene replacement, and generation of animal models for human diseases. The ability to isolate individual gene alleles will help to clarify whether a particular allele is associated with predisposition to different diseases, including cancer. Accumulated comprehensive knowledge of the genetic basis of human diseases provides a foundation for future medical research and has far-reaching implications for basic, clinical, and commercial efforts to understand, prevent, and treat diseases and develop new strategies for their diagnostic and treatment. The TAR technology is applicable to capture BGCs directly and efficiently from microbe organisms and environmental DNA samples, which are the source of the natural products for the pharmaceutical market. In addition, this will help us to understand BGCs biosynthesis and molecular mechanisms. TAR cloning is used to genetically engineer synthetic viruses with novel properties that may be used for the development of new vaccines. In perspective, given the potential of TAR-engineered HACs to deliver a TAR-cloned therapeutic gene(s) into cells with its associated regulatory elements offers a tremendous potential for gene therapy applications. Note that the HAC with the ability to carry an unlimited number of TAR-isolated genes [147] allows the development of multiple-gene humanized models, disease models, and the reprogramming and investigation of complex biomedical pathways.
